# Inhibition of experimental autoimmune encephalomyelitis by tolerance-promoting DNA vaccination focused to dendritic cells

**DOI:** 10.1371/journal.pone.0191927

**Published:** 2018-02-06

**Authors:** Timo Castor, Nir Yogev, Thomas Blank, Christina Barwig, Marco Prinz, Ari Waisman, Matthias Bros, Angelika B. Reske-Kunz

**Affiliations:** 1 Department of Dermatology University Medical Center, Mainz, Germany; 2 Institute for Molecular Medicine, University Medical Center, Mainz, Germany; 3 Institute of Neuropathology, Medical Faculty, University of Freiburg, Freiburg, Germany; 4 BIOSS Centre for Biological Signalling Studies, University of Freiburg, Freiburg, Germany; University of South Florida St Petersburg, UNITED STATES

## Abstract

In this study we analysed the effects of prophylactic biolistic DNA vaccination with plasmids encoding the encephalitogenic protein myelin oligodendrocyte glycoprotein (MOG) on the severity of a subsequently MOGp35-55-induced EAE and on the underlying immune response. We compared the outcome of vaccination with MOG-encoding plasmids alone or in combination with vectors encoding the regulatory cytokines IL-10 and TGF-ß1, respectively. MOG expression was restricted to skin dendritic cells (DCs) by the use of the DC-specific promoter of the fascin1 gene (pFscn-MOG). For comparison, the strong and ubiquitously active CMV promoter was employed (pCMV-MOG), which allows MOG expression in all transfected cells. Expression of IL-10 and TGF-ß1 was controlled by the CMV promoter to yield maximal synthesis (pCMV-IL10, pCMV-TGFß). Co-application of pFscn-MOG and pCMV-IL10 significantly ameliorated EAE pathology, while vaccination with pCMV-MOG plus pCMV-IL10 did not affect EAE outcome. In contrast, vaccination with either of the two MOG-encoding plasmids in combination with pCMV-TGFß significantly attenuated the clinical EAE symptoms. Mechanistically, we observed diminished infiltration of Th17 and Th1 cells as well as macrophages/DCs into the CNS, which correlated with decreased MOGp35-55-specific production of IL-17 and IFN-ϫ by spleen cells and reduced peptide-specific T cell proliferation. Our findings suggest deletion of or anergy induction in MOG-specific CD4^+^ T cells by the suppressive vaccination platform employed. MOG expression driven by the DC-specific fascin1 promoter yielded similar inhibitory effects on EAE progression as the ubiquitously active viral CMV promoter, when coapplying pCMV-TGFß. Our finding that pCMV-IL10 promoted tolerogenic effects only, when coapplied with pFscn-MOG, but not pCMV-MOG suggests that IL-10 affected only directly transfected DCs (pFscn-MOG), but not neighbouring DCs that engulfed MOG-containing vesicles derived from transfected keratinocytes (pCMV-MOG). Thus, due to its DC-restricted expression, the fascin1 promoter might be an interesting alternative to ubiquitously expressed promoters for vaccination strategies.

## Introduction

Multiple sclerosis (MS) is an inflammatory and demyelinating condition of the central nervous system (CNS), characterized by parenchymal infiltration of immune cells composed largely of T cells and macrophages [1]. Although the precise events that initiate MS remain unknown, numerous findings support the hypothesis that autoimmunity plays a major role in its pathology [2]. High similarities in terms of CNS immune cell infiltration, myelin destruction, neuronal death and subsequently paralysis as seen in MS patients, can be experimentally induced in laboratory rodents by immunization with CNS-derived antigens [3]. This form of disease induction, known as experimental autoimmune encephalomyelitis (EAE), is frequently used when attempting to study disease pathogenesis and testing innovative treatments. EAE is actively induced when an emulsion of myelin antigen like myelin oligodendrocyte glycoprotein (MOG) and a strong adjuvant (complete Freund´s adjuvant, CFA) is administered subcutaneously to naïve mice [4]. Hence, DCs may play a major role in the context of MS and its experimental model, as they shape the T cell repertoire, as well as activate and differentiate myelin-specific autoreactive T cells, which initiate disease pathology [5].

Current therapeutic strategies for MS use drugs that modify immune responses in general without specifically targeting the auto-aggressive T cells involved [6]. A therapeutic approach aimed at restoring tolerance to autoantigens is desirable. In this respect, generation of tolerogenic DCs that induce suppression of immune responses, is in the focus of research [7]. Isolation and manipulation of DCs ex vivo for therapeutic purposes, however, readily results in changes in phenotype and function, rendering in vivo manipulation of DCs an attractive goal.

DNA vaccination represents an antigen-specific approach to induce and shape cellular and humoral immune responses against plasmid-encoded antigens [8,9]. DNA vaccination also has the capacity to restore self-tolerance to a pathogenic autoantigen by means of clonal deletion of or induction of anergy in autoreactive T cells, immune deviation to an anti-inflammatory Th2 response or the induction of regulatory T cells (Tregs) [10]. In most instances naked plasmid DNA is administered either intramuscularly by needle injection or intradermally using the gene gun. For the biolistic application route via the gene gun, plasmid DNA is non-covalently coated onto gold microparticles, which are injected by helium pressure into the epidermal and dermal layers of the skin [11]. Because the particle-adsorbed plasmid is delivered directly into the cytosol and nucleus of cells, degradation is negligible. Up to 100 times lower amounts of DNA are required to yield similar effects, when this particle-mediated epidermal delivery (PMED) is compared with i.m. needle injection [12].

DNA vaccines to treat EAE encoded for TCR peptides [13] or different myelin proteins/peptides with and without immunomodulatory cytokines or other mediators (reviewed in [10]), and were in most instances applied by i.m. injection. A number of preclinical studies demonstrated amelioration or prevention of EAE by DNA vaccination [14], while in other studies the clinical symptoms exacerbated [15]. Clinical studies have demonstrated that DNA vaccines are well tolerated and have a good safety profile. Although phase I and II studies have shown some effects (reviewed in [10]), further improvements are required.

In most model systems, the strong and ubiquitously expressed viral CMV promoter was employed as a regulatory unit to control transgene expression [16]. Hence the transgene is expressed in all cells transfected after DNA vaccine application. A DNA vaccination strategy that specifically tolerizes DCs in combination with processing and presentation of a relevant (auto)antigen is desirable. This can be achieved by employing expression constructs under transcriptional control of a DC-focused promoter. The promoter of the cytoskeletal actin-bundling protein fascin1 was shown by us to drive transgene expression specifically in cutaneous DCs, but not in keratinocytes or dermal fibroblasts, when the skin of mice is biolistically transfected [17]. As we reported previously, biolistic DNA immunization of mice with plasmids encoding a transgene under control of the fascin1 gene promoter induced a strong systemic Th1 response, while plasmids controlled by the CMV promoter elicited a mixed Th1/Th2 response [18]. Both types of plasmid induced large numbers of transgene-specific CD8^+^ T cells producing IFN-ϫ. Restricting expression of a vector-encoded allergen to DCs was sufficient to convey inhibition of IgE production both prophylactically and therapeutically [19].

In the present work we used DC-specific biolistic gene transfer to generate tolerogenic DCs in order to inhibit subsequent induction of EAE. We used a plasmid encoding MOG under control of the fascin1 gene promoter (pFscn-MOG) together with vectors encoding the immunosuppressive cytokines IL-10 or TGF-ß1 as molecular adjuvants. Both cytokines were shown earlier to induce a tolerogenic state in DCs and T cells [20,21]. Their expression was controlled by the CMV promoter (pCMV-IL10, pCMV-TGFß) to achieve maximal production. The MOG-encoding and the cytokine-encoding plasmids were mixed and co-absorbed onto gold particles. Thus after biolistic application to the skin, in case of fascin1 gene promoter-driven MOG expression, presentation of processed MOG peptides is restricted to directly transfected DCs, which at the same time produce the immunosuppressive cytokine efficiently due to the strong CMV promoter. For comparison, we employed a plasmid encoding MOG under CMV promoter control (pCMV-MOG).

## Materials and methods

### Mice

C57BL/6 and 2D2/Thy1.1 [22] mice were bred and maintained in the Translational Animal Research Center of the University Medical Center Mainz under pathogen-free conditions on a standard diet. The recommendations of the Guide for the Care and Use of Laboratory Animals by the National Institutes of Health (NIH Publications No. 8023, revised 1978) were followed. The Ethics Commission according to the German Animal Welfare Act (Landesuntersuchungsamt of the state Rhineland-Palatinate) approved the experiments in this study (Reference No. 23 177-07/G08-1-007). Mice were euthanized by carbon dioxide asphyxiation.

### Cells

The moue embryonic fibroblast cell line NIH3T3 was cultured in IMDM with 5% FCS (PAA, Cölbe, Germany), 1 mM sodium pyruvate, 2 mM L-glutamine, 100 U/ml penicillin, 100 μg/ml streptomycin (all from Sigma-Aldrich, Deisenhofen, Germany), and 50 μM ß-mercaptoethanol (Roth, Karlsruhe, Germany). Bone marrow-derived DCs (BM-DCs) were differentiated from bone marrow progenitors of C57BL/6 mice as first described by Scheicher et al. [23] and modified by Bros and co-workers [24].

### Vectors

pCDH1-MCS1 (SBI, Mountain View, CA) contains the ubiquitiously active CMV promoter. To replace the CMV promoter by the mouse fascin1 gene promoter [17], a cloning vector that contained the subcloned mouse fascin1 gene promoter (pZero2.1-Fscn) was digested with BamHI, which cuts at the 5´ end within the vector polylinker, and filled in using Klenow enzyme. Afterwards, the linearized vector was digested with NheI, and the excised fascin1 promoter was isolated. pCDH1-MCS1 was digested with BcuI which cuts 5´ of the CMV promoter, 5´-protruding ends were blunted as described, followed by digestion with NheI. The vector backbone was isolated and aligned with the fascin1 promoter sequence. The vector pCDH1-copGFP (SBI) was used as a vector control in subsequent experiments, termed pcopGFP. The generation of pCMV-MOG has been described [25]. pFscn-MOG was obtained by the same cloning strategy. The generation of pCMV-IL10 has been described in that study. To generate pCMV-TGFß, the mouse TGF-ß1 minigene was amplified from C57BL/6 spleen cDNA by PCR (TGFß1-s 5´-CTGCTGCTTTCTCCCTCAAC-3´, TGFß1-as 5´-GGGTGCAGGTGTCCTTAAAT-3´), and was cloned into expression vector pCI containing the CMV promoter (Promega, Heidelberg, Germany) as described for the generation of CMV-IL10. As a control vector, the EGFP expression cassette in pEGFP-N1 (Takara/Clontech, Mountain View, CA) was cloned into the expression vector pCI, termed pCMV-GFP. All generated expression vectors were validated by sequencing.

### Transfections

NIH3T3 cells were transfected with pCMV-IL10 and pCMV-TGFß, respectively, for 5 h using GenePORTER reagent (Genlantis, San Diego, CA). The supernatant was removed and replaced by the respective culture medium. Cells were cultured for 72 h before analysis by real-time PCR (see 2.5).

BM-DCs were transfected with plasmid DNA as described [25]. In brief, BM-DCs (5×10^6^ cells/ml) harvested on d8 of culture were suspended in Resuspension buffer (NanoEnTek, Pleasanton, CA), and plasmid DNA (100 μg/ml) was added. Aliquots of cell suspension (100 μl) were electroporated using the MicroPorator MP-100 (VWR Life Science, Erlangen, Germany) at optimized conditions (1,450 V, one pulse of 30 ms). Transfectants (10^6^) were transferred to wells of 24-well tissue culture plates (Greiner, Frickenhausen, Germany) containing 2 ml of BM-DC medium. Twenty-four h later, transfectants were stimulated with LPS (1 μg/ml) for 24 h, for use in subsequent T cell stimulation assays.

### Real time PCR

Total RNA was isolated from differentially transfected NIH3T3 cells using the RNeasy NucleoSpin RNAII Total RNA Isolation (Kit Machery-Nagel, Düren, Germany), and reverse-transcribed (iScript; Bio-Rad, Munich, Germany) as recommended. Primer pairs used to detect expression of IL-10 and TGF-ß1, and of the house-keeping gene ubiquitin C used for normalization were obtained from eurofins MWG Synthesis (Ebersberg, Germany). Primer sequences and the performance of real time PCR have been described [24].

### EAE model

EAE was induced and monitored as described [13]. To this end, one part of MOGp35-55 (5 mg/ml; Research Genetics, Huntsville, AL) was emulsified with five parts CFA (Difco Laboratories, Franklin Lakes, NY) containing 1% (v/w) M. tuberculosis (Difco Laboratories), and with four parts PBS. C57BL/6 mice (6–10 weeks old) were injected subcutaneously at the tail base with 100 μl of this formulation. At the same day and after 48 h, mice were injected intraperitoneally with 200 ng pertussis toxin (Sigma-Aldrich, Deisenhofen, Germany). The course of EAE development was monitored for up to 24d, and symptoms were scored daily.

### Biolistic DNA vaccination

Biolistic transfection was performed as described [11] using the helium-driven Helios gene gun system (Bio-Rad, Munich, Germany). Plasmid DNA was purified from bacterial over-night cultures using the EndoFree Plasmid Maxi Kit (Qiagen, Hilden, Germany). Endotoxin levels were < 0.02 EU/μg DNA as assessed by *Limulus* amebocyte lysate assay (BioWhittaker, Walkersville, MD). Gold particles (Ø 1.6 μm) were coated with plasmid DNA, and cartridges were prepared as recommended by the manufacturer. Plasmids were used in equimolar amounts, using pcopGFP as reference (1μg/cartridge). Therefore, each cartridge contained about 2 μg of plasmid DNA precipitated onto 1 mg of gold particles. Cartridges were stored at 4°C for up to 2 weeks. Mice were immunized at a discharge pressure of 400 psi with two non-overlapping shots on shaved abdominal skin per type of plasmid DNA.

### Histology

Histological analysis was performed as described [26]. For this, spinal cords were removed on d16 after EAE induction, and a cranial part was fixed (4% buffered fomalin). Afterwards, spinal cords were embedded in paraffin. Sections were stained with luxol fast blue to monitor demyelination. Other sections were incubated with anti-macrophage-3 antigen (BD PharMingen) to identify macrophages and microglia, with anti-CD3 (T cells) and anti-B220 (B cells) (both from Serotec, Düsseldorf, Germany).

### Flow cytometry

Spleen and lymph node cells (each 2x10^6^), and CNS-derived cells prepared as described [27] (40% of volume), isolated from mice on d16 after EAE induction, were placed into wells of non-treated 96 well cluster plates and were washed in staining buffer (PBS/2% FCS). To block Fc receptor-mediated staining, cells were incubated with rat anti-mouse CD16/CD32 Ab (clone 2.4G2) purified from hybridoma supernantant, for 20 min on ice. Afterwards, cells were incubated with surface receptor-specific antibodies binding CD4 (clone RM4-5, labeled with PE-Cy5, CyChrome, or APC), CD11b (M1/70, FITC), CD45.2 (104, Biotin; detected using PE-labeled streptavidin), B220 (RA3-6B2, APC-Cy7), CD90.1 (HIS-51, PE), and CD25 (3C7, PE). For detection of the intracellular cytokines IFN-ϫ and IL-17A, cells were treated with 100 ng/ml PMA, 200 ng/ml Ionomycin, and 1 μg/ml Brefeldin A for 4 h, and washed in staining buffer. After incubation with antibodies specific for CD4 (RM4-5, APC), cells were permeabilized (Cytofix/Cytoperm™ Fixation/Permeabilization Kit, BD Biosciences) and incubated with cytokine-specific antibodies (IFN-ϫ: MXG1.2, FITC, and IL-17A: TC11-18H10, PE) as recommended. For staining of FoxP3 (FJK-16s, FITC) cells were permeabilized (Fixation/Permeabilization Kit, eBioscience) and treated as recommended. Appropriate isotype controls were employed. Samples were analyzed using a BD FACS Calibur flow cytometer equipped with CellQuest software (BD Biosciences). Data were analyzed using FlowJo software (FLOWJO, Ashland, OR).

### Proliferation assays

Differentially transfected BM-DCs (2x10^5^/ml) were seeded in 96 well cluster plates (100 μl; Corning, Cambridge, MA), and were serially diluted in triplicates. 2D2/Thy1.1 T cells (8×10^4^/100 μl) enriched from splenocytes as described [25] were added. To assess vaccination-induced T cell proliferation, spleen cells (5x10^5^) obtained from mice on d16 after EAE induction were cultured w/o or with titrated concentrations of MOGp35-55 in 0.2 ml volume in triplicates. T cell proliferation was assessed after 96 h of culture using ^3^H thymidine (0.25 μCi/well) applied for the last 16 h of culture. Cells were harvested onto glass fiber filters, and retained radioactivity was measured in a ß counter (1205 Betaplate, LKB Wallac, Finland).

For in vivo T cell proliferation assays, CD4^+^CD25^-^ cells were enriched by magnetic cell sorting (Miltenyi, Bergisch Gladbach, Germany) from 2D2/Thy1.1 spleen cells (CD90.1) as recommended by the manufacturer. Cells (2x10^7^/ml) were labeled with CFSE (2 μM) for 10 min. CFSE-labeled cells (10^7^ in 200 μl PBS) were transferred i.v. via the tail vain into C57BL/6 mice (CD90.2). On the next day, mice were immunized by biolistic transfection (see 2.7). Five days later, inguinal lymph nodes of immunized mice were retrieved, and cell suspensions were analyzed for proliferation of CFSE-labeled CD90.1^+^CD4^+^ T cells by flow cytometry (see above).

### Cytokine production by splenocytes

To assess MOGp35-55-specific cytokine production, spleens were isolated from mice on d16 after EAE induction. Spleen cells (5x10^6^) were seeded into wells of 24 well cluster plates and were cultivated w/o or with MOGp35-55 (25 μg/ml) in a volume of 2 ml for 96h. Contents of cytokines in supernatants were quantified using a sandwich-ELISA as previously described [28]. ELISA capture (IFN-ϫ: R4-6A2, IL-17: 50101) and biotinylated detection antibodies (IFN-ϫ: AN18.17.24, IL-17: goat polyclonal) were used as recommended by the manufacturer (R&D Systems, Wiesbaden, Germany). The according recombinant murine cytokines were used as standards.

### Data analysis

Data are presented as means ± SEM of the values. Data were analyzed for statistically significant differences by applying Student´s t test.

## Results

### In vitro and in vivo expression of MOG-encoding constructs

**Fig 1:**
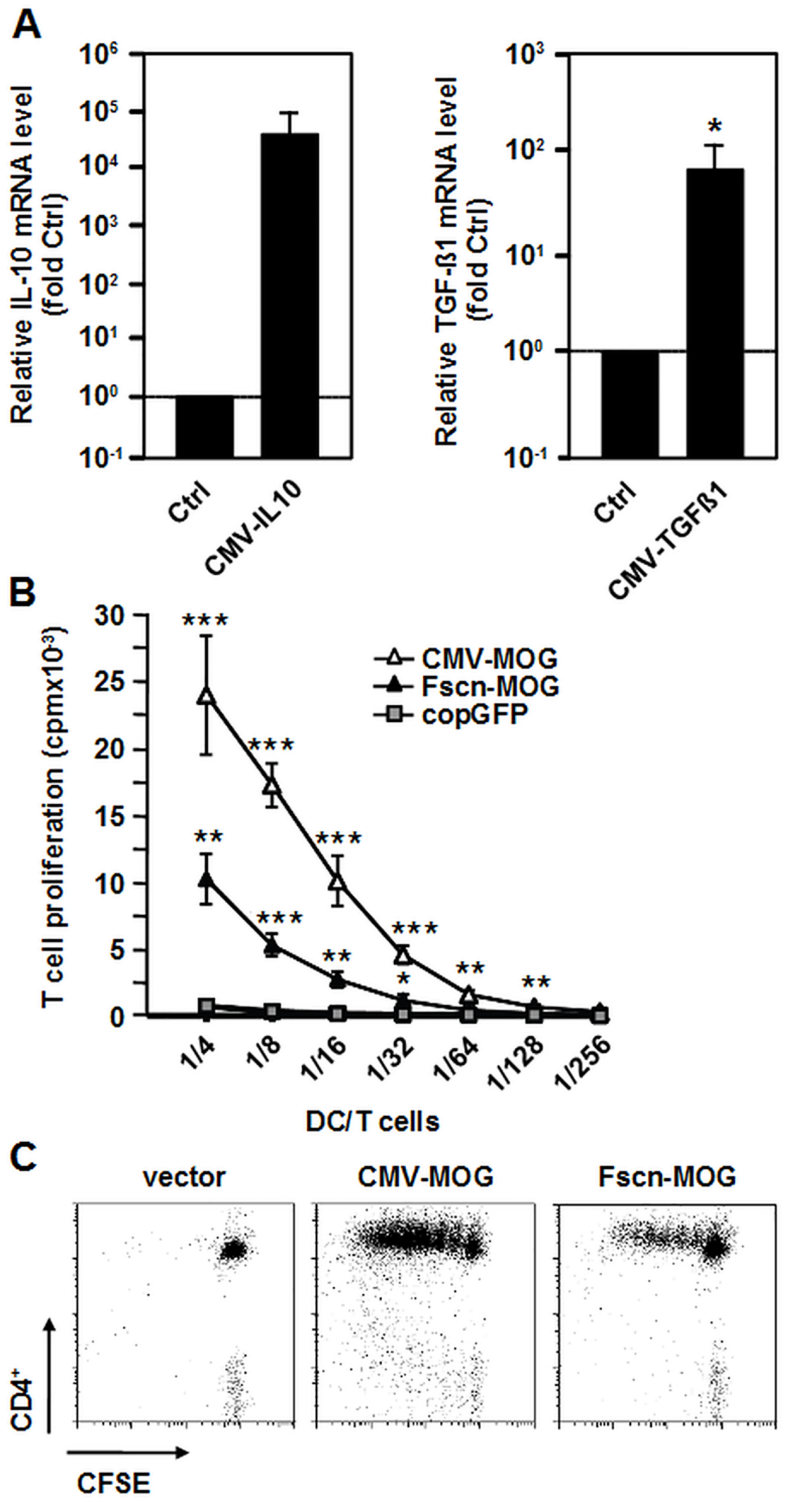
Functional analysis of cytokine and MOG expression constructs in vitro and in vivo. (A) Quantitative RT-PCR was performed 72 h after transfection of NIH-3T3 cells with pCMV-IL10 or pCMV-TGFß. N = 4 independent experiments. (B) BM-DCs (day 8) were control transfected (copGFP) or were transfected with the MOG-encoding plasmids in equimolar amounts and were stimulated 1 day later with LPS for 24 h. Titrated numbers of cells were cultured with 8x10^4^ T cells from 2D2/Thy1.1 mice for 96 h in triplicates and proliferation was determined by ^3^H-thymidine incorporation. Representative of N = 3 independent experiments. (C) MACS-purified CFSE-labeled CD4^+^CD25^-^ T cells from 2D2/Thy1.1 mice (CD90.1^+^) were adoptively transferred into CD90.2^+^ C57BL/6 mice 24 h before DNA vaccination with equimolar amounts of DNA. Five days after vaccination, cytofluorometric analysis of CD90.1^+^CD4^+^ cells in mesenteric lymph nodes was performed. The gating strategy for analysis of adoptively transferred T cells is depicted in S1 Fig. Representative of N = 3 independent experiments. *p<0.05, **p<0.01, p<0.001 versus non-transfected/control-transfected cells.

To protect mice via biolistic DNA vaccination from EAE development, induced by their treatment with MOGp35-55 peptide/CFA and pertussis toxin, we generated MOG-encoding expression plasmids and plasmids encoding the immunoregulatory cytokine TGF-ß1 and IL-10, respectively, which were reported earlier to induce a tolerogenic state in DCs and T cells [20,21]. Expression of MOG was controlled either by the fascin1 promoter (pFscn-MOG), the expression of which in the skin is restricted to DCs [17], or by the strong and ubiquitously active CMV promoter (pCMV-MOG). Antigen specificity was controlled by use of a vector encoding copGFP under the control of the CMV-promoter (pcopGFP). Expression of TGF-ß1 and IL-10, respectively, was under control of the CMV promoter (pCMV-TGFß, pCMV-IL10) to allow for efficient transgene expression. The corresponding control construct coded for EGFP under CMV-promotor control (pCMV-GFP). Functional activity of the two cytokine encoding vectors was analysed by quantitative PCR after transfection of NIH-3T3 cells used as a well-transfectable cell line. IL-10 transcripts increased by a factor of ca. 37,800 in transfected versus untransfected cells, while TGF-ß1 mRNA increased ca. 57-fold (Fig 1A).

Next, we determined the potency of the two MOG-encoding vectors to transfect BM-DCs. Twenty-four h after transfection, the cells were matured in the presence of LPS for an additional 24 h and were cocultured with T cells from 2D2/Thy1.1 mice. Both transfected DC populations induced 2D2/Thy1.1 T cell proliferation (Fig 1B). As expected, BM-DCs transfected with pCMV-MOG were better stimulators for 2D2/Thy1.1 T cells than were cells transfected with pFscn-MOG. However, the T cell stimulating potency of the latter DC population was only 4 times lower than that of BM-DCs transfected with pCMV-MOG. This finding corroborates our earlier results, showing a higher transcriptional activity of the fascin1 promoter in maturing DCs relative to other DC-specific promoters like the dectin-2 promoter [17]. T cell proliferation was MOG-specific, because transfection with the control vector pcopGFP, encoding copGFP instead of MOG, did not result in T cell stimulatory potency of BM-DCs.

We also tested, whether biolistic immunization of mice with pFscn-MOG or pCMV-MOG yielded MOGp35-55 presenting APCs in the draining lymph nodes. For this, carboxyfluorescein succinimidyl ester (CFSE)-labeled naïve CD4^+^CD25^-^ T cells from 2D2/Thy1.1 (CD90.1^+^) mice were adoptively transferred to C57BL/6 recipients (CD90.2^+^). On the next day, these were biolistically transfected with pCMV-MOG, pFscn-MOG or with the empty control vector (pCDH1-MSC1) in equimolar amounts via the gene gun. Five days later, proliferation of CFSE-labeled 2D2/Thy1.1 T cells within the population of inguinal lymph node cells was determined by cytofluorometry. Application of both MOG-encoding constructs yielded 2D2/Thy1.1 T cell proliferation in contrast to the control vector, demonstrating antigen specificity of the response (Fig 1C). These findings show that following biolistic transfection with the two MOG-encoding plasmids, transfected DCs have migrated from the skin to the draining lymph nodes resulting in presentation of MOG peptides with ensuing activation of 2D2/Thy1.1 T cells, which is in accordance with earlier reports [29]. Again, as expected, more 2D2/Thy1.1 cells proliferated, when the strong and ubiquitously active CMV promoter instead of the fascin1 promoter was employed, while the number of detected T cell cycles was comparable. In case of biolistic vaccination with pCMV-MOG, all transfected cells (i.e. keratinocytes, DCs etc.) can synthesize MOG. MOG peptides can be presented by directly transfected DCs, but also by non-transfected DCs, which have phagocytosed apoptotic vesicles released from transfected apoptotic keratinocytes [30]. Therefore, after biolistic application of pCMV-MOG, the number of MOG-presenting DCs and subsequently the T cell proliferative response induced is higher as compared with biolistic transfection with pFscn-MOG.

### Prophylactic biolistic coapplication of MOG- and TGF-ß1- or IL-10-encoding plasmids ameliorates clinical outcome of MOGp35-55-induced EAE

The two MOG-encoding plasmids as well as the plasmids encoding TGF-ß1 or IL-10 and the respective control plasmids were mixed in various combinations in equimolar amounts, and were adsorbed to gold particles, which were then biolistically applied to the skin of C57BL/6 mice for three times at weekly intervals. Seven days after the last vaccination, EAE was induced using MOGp35-55 peptide/CFA and pertussis toxin, and the clinical outcome was monitored.

**Fig 2:**
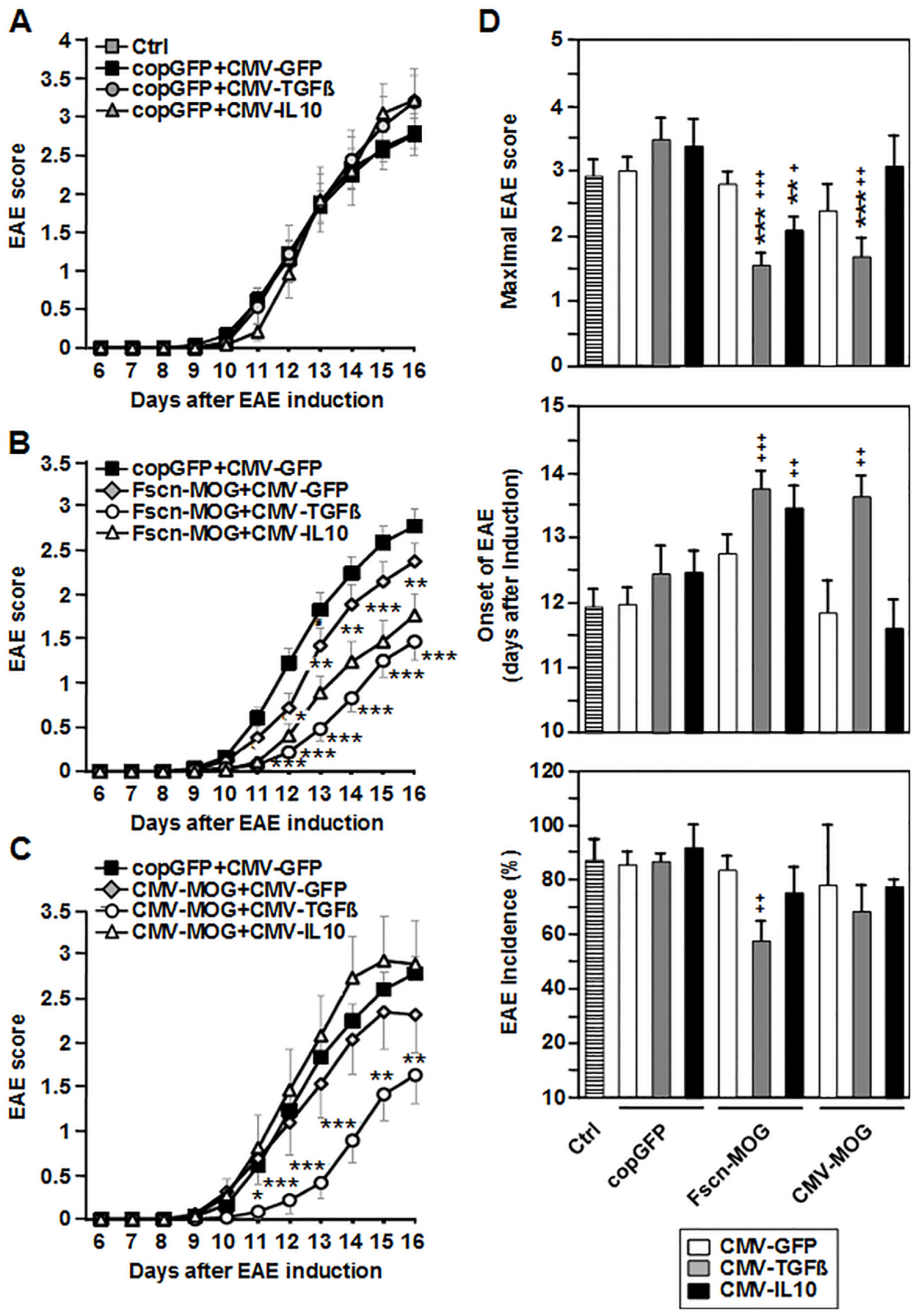
Biolistic coapplication of MOG- and cytokine-encoding plasmids ameliorates clinical outcomes of MOGp35-55-induced EAE. C57BL/6 mice were biolistically transfected with the indicated plasmid combinations in equimolar amounts 3 times in weekly intervals before induction of EAE. (A-C) Clinical symptoms were evaluated by EAE score for the following 16 days. For better clarity, data are shown in 3 graphs. Data of the reference group copGFP+CMV-GFP are the same in the 3 graphs. (D) Start of EAE symptoms, maximal EAE score and EAE incidence are depicted. N = 12 to 57 mice per group. *p<0.05, **p<0.01, ***p<0.001 versus copGFP+CMV-GFP mice, ^++^p<0.01, ^+++^p<0.001 versus non-vaccinated mice.

Mice cotransfected with the control vectors pcopGFP+pCMV-GFP exhibited similar EAE symptoms as did control mice that were not treated with plasmids, indicating that gene gun treatment per se did not influence EAE pathology (Fig 2A). Likewise, application of TGF-ß1- or IL-10-encoding vectors in the absence of MOG-encoding plasmids, but in the presence of the control vector (pcopGFP+pCMV-TGFß, pcopGFP+pCMV-IL10) did not modify EAE symptoms. Cotransfection of mice with pFscn-MOG+pCMV-GFP resulted in an only slight, non-significant reduction in EAE symptoms as compared with the control transfection with pcopGFP+pCMV-GFP (Fig 2B). However, a significant reduction in symptoms was attained by cotransfection with pFscn-MOG+pCMV-IL-10 or pFscn-MOG+pCMV-TGFß. Treatment of mice with the combination of pFscn-MOG+pCMV-TGFß+pCMV-IL10 did not result in stronger reduction of symptoms (data not shown). As shown in Fig 2C, cotransfection of mice with pCMV-MOG+pCMV-GFP did not alleviate EAE symptoms, while cotransfection with pCMV-MOG+pCMV-TGFß significantly reduced the symptom score. Notably, cotransfection with pCMV-MOG+pCMV-IL10 was not effective.

As depicted in Fig 2D, the maximal EAE score was significantly reduced and the onset of EAE symptoms was significantly delayed in those groups vaccinated with MOG under control of the fascin1 promoter together with TGF-ß1 or IL10 encoding plasmids, as well as in the group treated with MOG under control of the CMV promoter in combination with pCMV-TGFß. All other groups showed a maximal EAE score and onset of EAE symptoms that was comparable with the non-vaccinated control group. The EAE incidence was only significantly reduced in the Fscn-MOG+CMV-TGFß group. Due to the inefficiency of combined vaccination of mice with pCMV-MOG plus pCMV-IL10 to attenuate EAE symptoms, we omitted this group in subsequent experiments.

### Cell infiltration into the CNS is reduced and demyelinisation of axons is diminished following DNA vaccination

**Fig 3:**
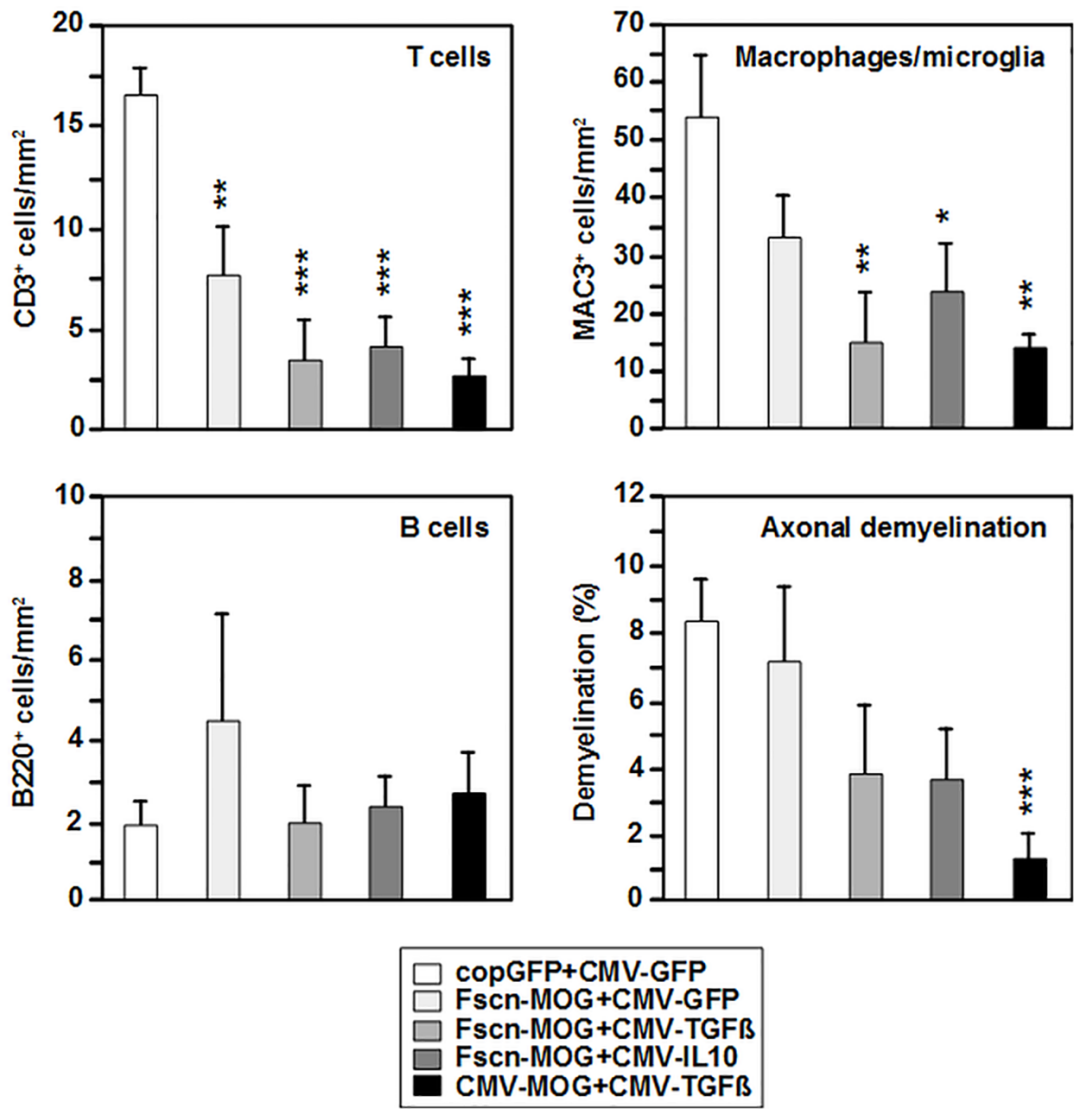
Biolistic co-application of MOG- and cytokine-encoding plasmids results in reduced cellular infiltration into cranial spinal cord as well as in diminished demyelination. Mice were vaccinated, and EAE was induced as described (see Fig 2). On d16 after EAE induction, the cranial part of the spinal cord was prepared, and sections were incubated with anti-CD3, anti-MAC3, and anti-B220 antibodies as well as with luxol fast blue to assess demyelination. Examples of stained sections are shown in S2 Fig. N = 5–7 mice per group. *p<0.05, **p<0.01, ***p<0.001 versus copGFP+CMV-GFP mice.

To analyse the impact of DNA vaccination on the infiltration of immune cells into the CNS and on the demyelination of axons, the cranial spinal cord of vaccinated mice was prepared on day 16 of EAE induction, when the clinical symptoms were maximally apparent. Histological analysis of sections of the cranial spinal cord revealed reduced numbers of CD3^+^ T cells and MAC-3^+^ macrophages/microglia in the spinal cord of mice cotransfected with pFscn-MOG plus the control vector pCMV-GFP in comparison with control mice, albeit below statistical significance in case of macrophages/microglia (Fig 3). In the spinal cord of mice vaccinated with pFscn-MOG in combination with pCMV-TGFß or pCMV-IL10 or with pCMV-MOG+pCMV-TGFß even less T cells and macrophages/microglia were apparent. This finding suggests reduced infiltration of T cells and macrophages into the spinal cord of these groups of mice. In mice, which were vaccinated with MOG-encoding plasmid in combination with either IL-10- or TGF-ß1-encoding vector reduced axonal demyelinisation was observed. This reduction was only significant, when mice were cotreated with pCMV-MOG and pCMV-TGFß. There was no reduction in the number of infiltrating B220^+^ B cells.

**Fig 4:**
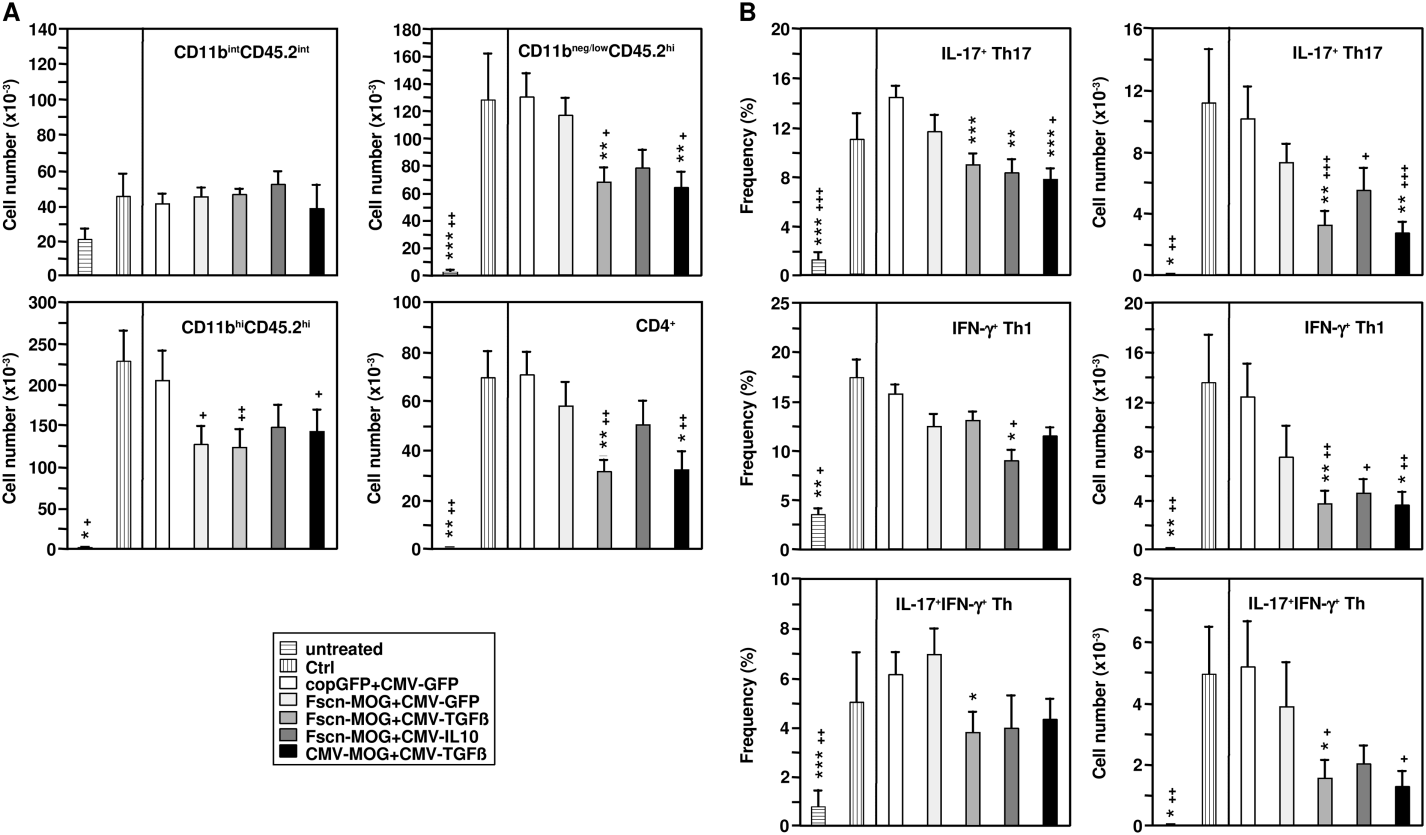
Biolistic coapplication of MOG- and cytokine-encoding plasmids attenuates infiltration of Th1 and Th17 into the CNS after EAE induction. Mice were vaccinated, and EAE was induced as described (see Fig 2). On d16 after EAE induction, brains and spinal cords were prepared, and cells were enriched by Percoll density centrifugation. (A) Immune cell populations were identified by antibody staining to delineate infiltrating myeloid cell populations and T cells using according lineage markers. The gating strategy is depicted in S3 Fig. Abbreviations: int, intermediate, hi, high. (B) Isolated cells were treated for 4h with PMA (100 ng/ml), Ionomycin (200 ng/ml) and Brefeldin A (1 μg/ml), followed by detection of surface CD4, and intracellular detection of cytokines. The gating strategy is depicted in S4 Fig. (A) N = 5–21 and (B) N = 5–16 mice per group. *p<0.05, **p<0.01, ***p<0.001 versus copGFP+CMV-GFP mice, ^+^p<0.05, ^++^p<0.01, ^+++^p<0.001 versus Ctrl mice.

In addition, we performed cytofluorometric measurements of cellular infiltration into the brain and spinal cord. Microglia and cellular infiltrates were enriched by Percoll gradient density centrifugation. Cells were stained with antibodies directed to CD4, CD11b, and CD45.2. The CNS-resident microglia cells were present in roughly similar numbers in all groups of mice (Fig 4A). Following EAE induction in non-vaccinated mice as well as in control vector-treated mice a strong infiltration of CD11b^-/low^CD45.2^high^ cells (T cells, B cells, DCs), of CD11b^high^CD45.2^high^ cells (macrophages, DCs), and of CD4^+^ T cells was noticed as compared with untreated control mice not subjected to EAE induction. Infiltration of these cell populations was reduced in all groups cotreated with MOG- plus cytokine-encoding vectors, albeit without reaching significance in case of mice vaccinated with pFscn-MOG+pCMV-IL-10. Reduction of cellular infiltration was more pronounced, when the immunomodulator TGF-ß1 instead of IL-10 was employed irrespective of the promoter used to drive MOG expression.

The infiltrated CD4^+^ T cells were further characterized with respect to their production of IL-17 and IFN-ϫ by intracellular staining, following stimulation of the cells by PMA, Ionomycin and Brefeldin A. Cytofluorometric analysis revealed a significant reduction in the percentage of Th17 cells in the groups Fscn-MOG+CMV-TGFß, Fscn-MOG+CMV-IL10, and CMV-MOG+CMV-TGFß in comparison with the control vector-treated group (copGFP+CMV-GFP) (Fig 4B, upper left). There was also a slight reduction in the percentage of Th1 cells in these groups, although this was only significant in the Fscn-MOG+CMV-IL10 group (Fig 4B, middle left). Infiltration of IL-17^+^IFN-ϫ^+^ Th cells was also diminished in these three groups of mice (Fig 4B, lower left). When the total number of Th17, Th1 and double positive Th cells in the brain and spinal cord was calculated, a significant reduction was evident in all three groups of mice (Fig 4B, right panels). The extent of T effector cell reduction was comparable when TGF-ß was coexpressed with MOG under control of either the fascin1 or the CMV promoter.

### Improvement in EAE severity correlates with decreased IL-17 and IFN-ϫ production as well as diminished T cell proliferation in spleen

**Fig 5:**
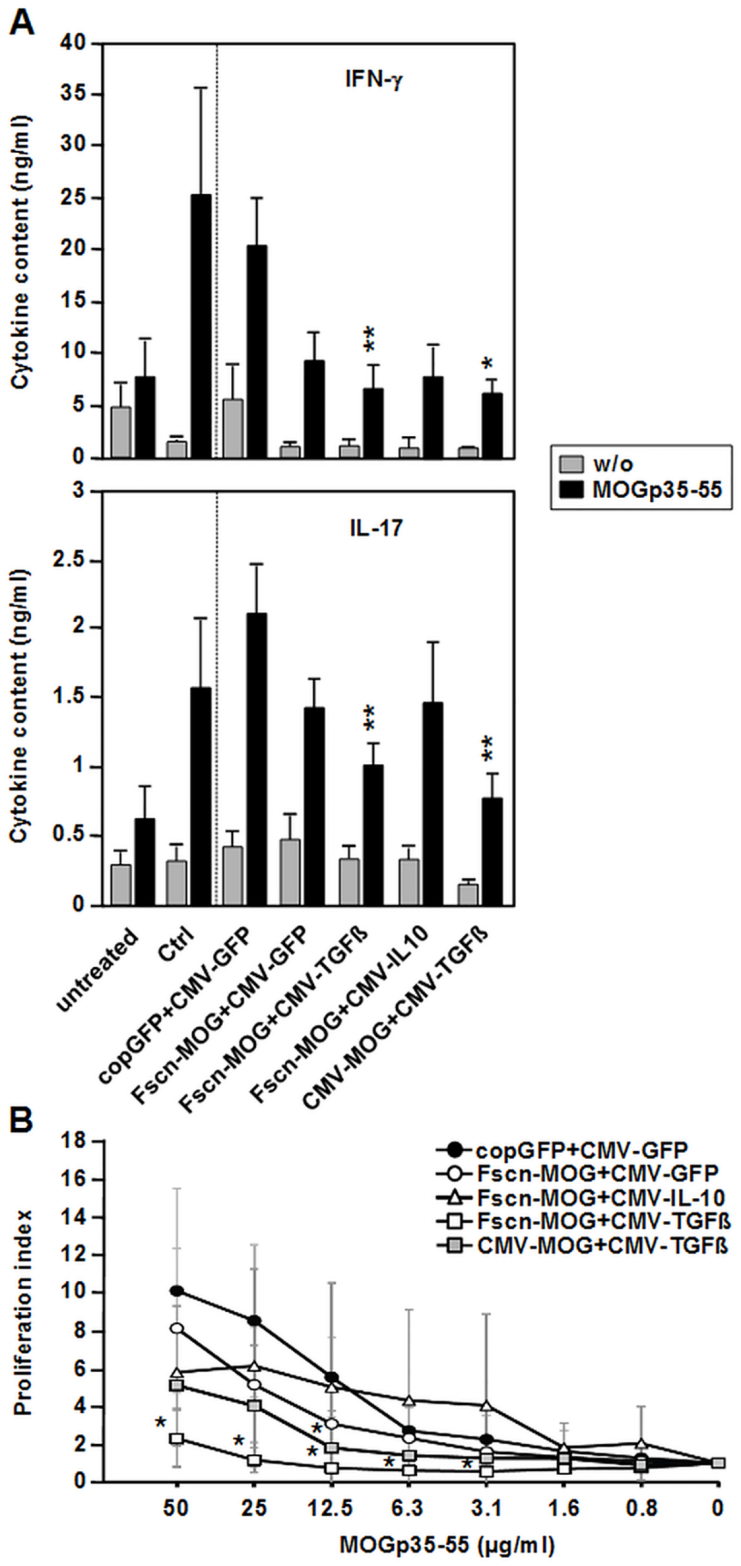
Spleen cells derived from mice vaccinated with MOG- and cytokine-encoding plasmids show attenuated proliferation and IL-17/IFN-ϫ production in response to restimulation. Mice were vaccinated, and EAE was induced as described (see Fig 2). On d16 after EAE induction, spleen cells were retrieved. (A) Spleen cells (5x10^6^/2 ml/well) were cultured w/o (grey bars) or with 25 μg/ml MOGp35-55 peptide (black bars) for 96h, and supernatants were harvested for cytokine measurements. N = 6–15 mice per group. (B) Spleen cells (5x10^5^/well) were cultured in triplicates w/o or with MOGp35-55 peptide at the concentrations indicated for 96h, and proliferation was assessed by incorporation of ^3^H-thymidine for the last 16 h of culture. Proliferation index was calculated by dividing the mean of antigen-specific proliferation by the corresponding mean of unspecific proliferation. N = 4–5 mice per group. *p<0.05, **p<0.01, versus copGFP+CMV-GFP treated mice.

We next analysed, whether the systemic Th response was altered by DNA vaccination of mice followed by EAE induction. Spleen cells recovered on day 16 after the start of EAE induction were cultivated in the presence and absence of an optimal dose of MOGp35-55 peptide for four days. The contents of IL-17 and IFN-ϫ in cell culture supernatants were then determined by ELISA. In supernatants of spleen cells isolated from mice without any treatment, no MOGp35-55-specific IL-17 or IFN-ϫ production was found (Fig 5A). Induction of EAE in non-vaccinated mice and in mice treated with the control vectors pcopGFP+pCMV-GFP yielded significant secretion of both cytokines. In comparison with the latter group, reduced levels of the two cytokines were detected in the groups vaccinated with pFscn-MOG and pCMV-GFP or together with pCMV-IL10, however without reaching statistical significance. In contrast, vaccination with either of the two MOG-encoding vectors along with pCMV-TGFß significantly diminished both cytokines at a comparable extent, which confirmed our previous finding that in case of coapplication of pCMV-TGFß no difference between fascin1 and CMV promoter-driven MOG expression was observed.

To assess the antigen-specific proliferative potential of Th cells, splenocytes from various groups of mice were cultured in the presence and absence of titrated doses of MOGp35-55 peptide for four days and proliferation was determined by incorporation of ^3^H-thymidine into cellular DNA. Mice treated with the control vectors before induction of EAE proliferated efficiently in response to MOGp35-55 peptide stimulation (Fig 5B). Antigen-specific proliferation was significantly inhibited in pFscn-MOG+pCMV-TGFß vaccinated mice over the whole range of peptide doses employed and less so in pCMV-MOG+pCMV-TGFß treated mice. In spleen cells from mice treated with pFscn-MOG plus either pCMV-GFP or pCMV-IL10, inhibition of the proliferative response was not significantly affected.

### DNA vaccination does not induce regulatory T cells or a shift towards a Th2 response

**Fig 6:**
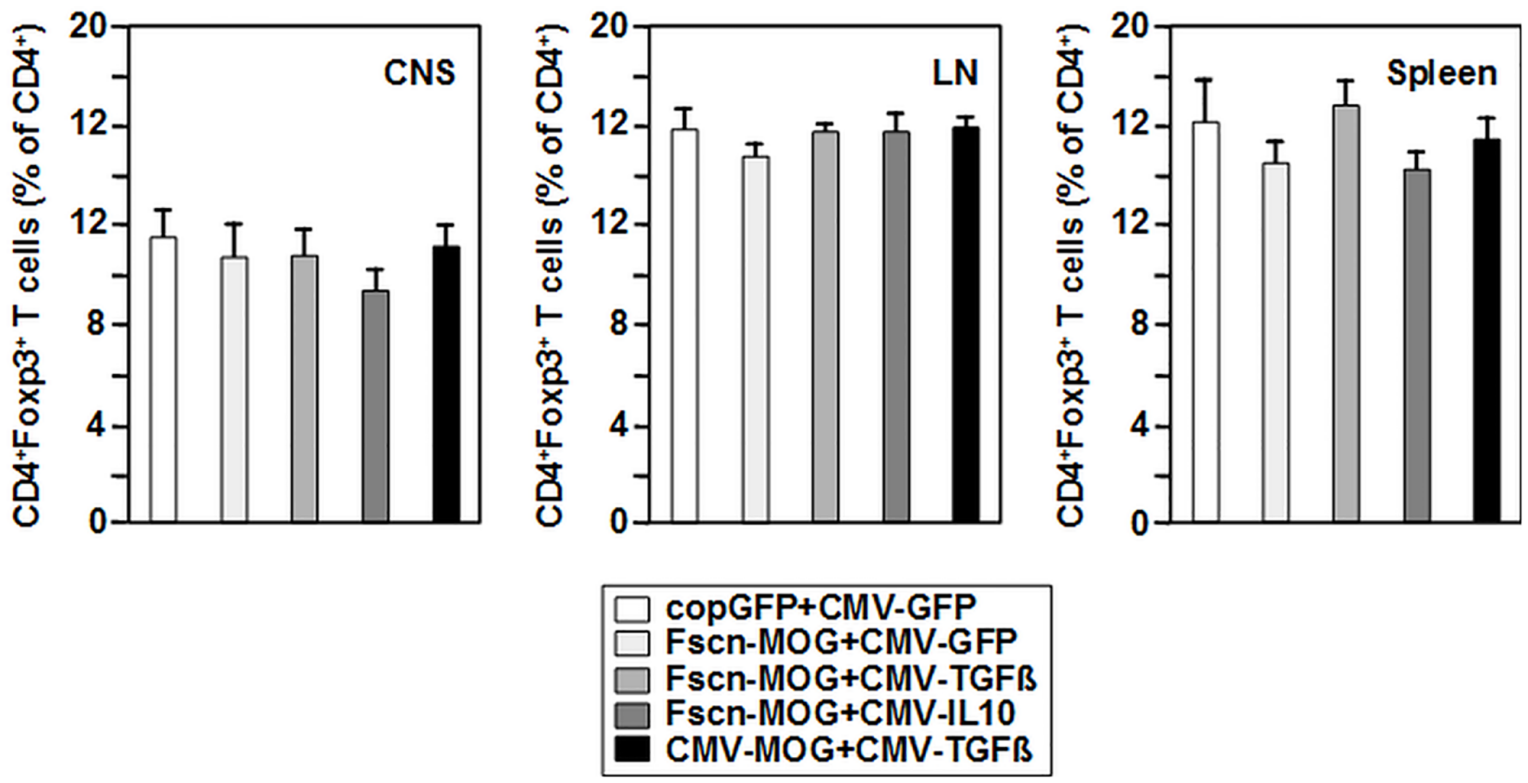
Preventive vaccination of mice with MOG- and cytokine-encoding plasmids has no effect on the frequencies of CD4^+^Foxp3^+^ T cells in CNS and lymphoid organs after EAE induction. Mice were vaccinated, and EAE was induced as described (see Fig 2). On d16 after EAE induction, brain and spinal cord, inguinal lymph nodes and spleen were prepared, and frequencies of CD4^+^Foxp3^+^ T cells were assessed by flow cytometry. The gating strategy is depicted in S5 Fig. N = 10–21 mice per group.

The finding of a reduced T cell proliferative capacity of the peptide-restimulated spleen cells suggests deletion of MOG-reactive T cell clones, the induction of anergy in the MOG-specific T cell population or increased activity of Tregs. To investigate the involvement of Tregs, we determined the fraction of FoxP3^+^ Tregs with or without CD25 expression within the CD4^+^ T cell population in spleen, inguinal lymph nodes and CNS/spinal cord, isolated during the peak of disease, by cytofluorometry. Neither in the lymphoid organs nor in the brain and spinal cord, the sites of inflammation, significant differences in the percentage of CD4^+^FoxP3^+^ Tregs were detectable (Fig 6). This holds true for the fraction of CD4^+^FoxP3^+^CD25^+^ as well as CD4^+^FoxP3^+^CD25^-^ Tregs (data not shown). Along this line, we found no major differences between the groups, when the levels of the two suppressive cytokines IL-10 and TGF-ß was determined in culture supernatants of spleen cells cultured with or without an optimal dose of MOGp35-55 peptide for four days (data not shown). This finding also argues against the development of FoxP3^-^ Tr1 cells. In addition, the level of IL-5 production as a marker of Th2 cells was determined. We did not observed peptide-specific IL-5 secretion (data not shown).

Taken together, these results revealed that Tregs were not induced by the DNA vaccination regime used. Also, the amelioration in EAE symptoms did not depend on a shift in immune response towards Th2 cells. Our findings suggest deletion of or anergy induction in MOG-specific CD4^+^ T cells by vaccination with MOG-encoding vectors combined with the TGF-ß1-encoding plasmid as the basis of inhibition of EAE pathology. Hereby the DC-specific fascin1 promoter yielded similar results as the ubiquitously active viral CMV promoter.

## Discussion

The extent of cell infiltration into the CNS correlates with the severity of clinical EAE symptoms [31]. Especially Th17 and Th1 cells are critically involved in the initiation and progression of EAE pathology [32]. Following activation and differentiation in the periphery these T cell populations migrate to the CNS, where they are reactivated antigen-specifically by CNS-resident APCs leading to an inflammatory response with ensuing progressive pathology [33,34].

Studies in the EAE model as well as with MS patients have shown that i.m. administration of myelin antigen-encoding plasmids can result in the induction of tolerance, which ameliorates disease symptoms, although there have been conflicting results (reviewed in [10]). To the best of our knowledge, only one report is available in the literature describing the effects of DNA vaccination applied via gene gun on EAE progress. In this regard, Ramshaw et al. [35] observed improved disease development following biolistic vaccination of rats with MBP-encoding plasmid DNA, which was accompanied by a shift towards a Th2 response. Interestingly, i.m. needle injection of the same plasmid had no effect. Indeed, gene gun vaccination was suggested before to favour a Th2 cytokine pattern [36,37]. We reported previously, that antigen-encoding plasmids controlled by the CMV promoter elicited a mixed Th1/Th2 response, when biolistically applied to mice, while plasmids encoding a transgene under control of the fascin1 gene promoter induced a systemic Th1 response [18]. Accordingly, in our study described here we did not detect enhanced production of IL-5 by splenocytes used as a Th2 marker cytokine in groups of mice vaccinated with the different MOG- and cytokine-encoding vectors (data not shown). This difference might be explained by variations in the helium discharge pressure, since increased pressure was reported to augment Th1-polarisation [38].

In most of the studies reported up to now, expression of the encephalitogenic antigen was controlled by the ubiquitously active CMV promoter, which allows for antigen expression in all transfected cells. In our study we employed the promoter of the fascin1 gene to focus expression of MOG specifically to DCs. The complete MOG gene was used for DC transfection to address diverse T cell clones recognizing different epitopes within the MOG protein. Gene gun-mediated vaccination of mice with pFscn-MOG or pCMV-MOG alone yielded only moderate inhibitory effects on the progression of subsequently induced EAE. On the other hand, Fissolo et al. [39], Jégou et al. [14] and Walczak et al. [40] demonstrated significant reduction of clinical symptoms of EAE in C57BL/6 mice vaccinated i.m. with MOG-encoding DNA. The different outcome might be based on the different route of plasmid application (gene gun versus i.m. injection). It is also possible that the length of the time period passed between the last DNA application and induction of EAE is essential for vaccination success, being 7 days in our experiments and 14 and 28 days, respectively, in the three reports cited above. In contrast, Bourquin et al. [15] reported that i.m. vaccination of SJL mice with MOG-encoding DNA resulted in an exacerbated form of EAE, which was associated with a pathogenic MOG-specific autoantibody response, which might be due to differences in the mouse strains.

In order to render MOG-presenting DCs tolerogenic, we employed the cytokines IL-10 and TGF-ß1, respectively, shown in many studies to induce a tolerogenic state in DCs and T cells acting in an autocrine and paracrine manner [20,21]. We used these two well-characterized anti-inflammatory and immunosuppressive cytokines rather than ambiguous cytokines like e.g. GM-CSF, which was reported to exert pro-inflammatory function in some experimental settings, but nevertheless was also shown to suppress autoimmune diseases indirectly by mediating tolerance via inducing tolerogenic DCs, probably depending on the dose of GM-CSF [41–44]. Expression of IL-10 and TGF-ß1 was controlled by the strong CMV promoter in order to attain efficient cytokine synthesis. We hypothesized that in mice vaccinated with pFscn-MOG only directly transfected DCs express MOG. Because these DCs co-express the inhibitory cytokine, they are rendered tolerogenic by an autocrine effect. On the other hand, when pCMV-MOG is biolistically applied, most of the transfected cells are keratinocytes with DCs also transfected. Thus, MOG peptides are not only presented by directly transfected DCs, but also by non-transfected DCs, which have phagocytosed apoptotic vesicles released from transfected apoptotic keratinocytes [45]. These non-transfected DCs, which nevertheless present MOG-peptides, do not (over)express the inhibitory cytokine, and are therefore not tolerized. Thus, they may act as mature APCs, counteracting the inhibitory effect of the directly tolerized DCs. According to our hypothesis the balance between the developing tolerogenic and immunogenic MOG-presenting DCs is critical for the outcome of vaccination.

Our results obtained with preventive biolistic co-application of either of the two MOG-encoding vectors and pCMV-IL10 appear to corroborate this hypothesis. As predicted by our hypothesis, co-application of pCMV-MOG and pCMV-IL10 did not affect EAE outcome. In contrast, co-application of pFscn-MOG and pCMV-IL10 significantly ameliorated EAE pathology associated with a delayed onset of EAE symptoms and a reduced EAE score. This effect was antigen-specific and antigen-dependent, because the respective control plasmids did not exert a significant inhibitory effect. The immunoregulatory cytokine IL-10 inhibits T cells and APCs in vitro and in vivo [46]. Either directly or indirectly via DCs, IL-10 induces the generation of anergic T cells and Tregs. DCs treated in vitro with recombinant IL-10 [47] or transfected in vitro with an IL-10-encoding plasmid [48] were reported to ameliorate subsequently induced EAE following transfer into recipients.

In contrast, vaccination of mice with either of the two MOG-encoding plasmids in combination with pCMV-TGFß as an alternative inhibitory cytokine-encoding vector significantly ameliorated the clinical EAE symptoms and resulted in diminished infiltration of Th17, Th1, IL-17/IFN-ϫ double-positive cells and macrophages/DCs into the CNS as compared with control-vaccinated mice. However, while expression of MOG driven by the CMV or the fascin1 promoter in combination with pCMV-TGFß resulted in a clear reduction of CNS-infiltrating macrophages and T cells (see Fig 3; S2 Fig) as well as Th1/Th17 T effector cell populations (see Fig 4), axonal demyelination was significantly reduced only in the case of CMV promoter-driven MOG expression. Further studies are required to delineate this difference.

In agreement with the pronounced EAE preventing effects of cotransfected pCMV-TGFß, DCs treated with TGF-ß protein were reported to suppress immune responses and to inhibit EAE pathology [49]. Our results show that endogenous production of TGF-ß1 by in vivo transfected DCs is efficient in ameliorating EAE symptoms and the infiltration of immune cells into the CNS both when using a DC-specific (fascin1) or ubiquitously active (CMV) promoter to drive MOG expression. These findings correlated with decreased production of IL-17 and IFN-ϫ by spleen cells as well as with a reduced proliferative response of restimulated splenocytes. These effects were MOGp35-55-specific, demonstrating antigen-specific inhibition of Th17 and Th1 immune responses by the preventive DNA vaccination platform used. TGF-ß was shown before to inhibit generation of Th1 (and Th2) cells via suppression of T-bet (and GATA3), while it supported differentiation of Th17 and FoxP3^+^ Treg cells [21]. Therefore, we expected that the reduced level of Th17 and Th1 cells in the CNS and the periphery following DNA vaccination correlated with augmentated numbers of FoxP3^+^ Tregs. However, no elevation in the percentage of FoxP3^+^ Tregs within the CD4^+^ T cell population in CNS, spleen and lymph nodes was detectable. Because the number of CD4^+^ T cells infiltrating into the CNS was reduced after vaccination with MOG-encoding plus cytokine-encoding plasmids, the total number of CD4^+^FoxP3^+^ Tregs in the CNS was decreased as well. However, flow cytometric measurements of FoxP3^+^ Tregs were performed in an antigen-unspecific context. Thus, it is not clear, whether quantitative differences might exist in the number of MOG-reactive Tregs. However, TGF-ß1 and IL-10 production by MOG-activated spleen cells which may be generated predominantly by FoxP3^+^ and by Tr1 Tregs, was also not augmented. Thus, mechanisms other than an enhancement of Tregs seem to be active following DNA vaccination in the inhibition of the Th17 and Th1 response.

Along this line, in a model of oral tolerance induction, it was demonstrated that antigen given in a high dose led to anergy induction, while low-dose antigen yielded active suppression [50]. It might be possible that in vivo transfected DCs arising following application of MOG-encoding plasmids via gene gun present the cognate peptide/MHC complexes at a high frequency that yields induction of anergy in interacting T cells rather than promoting the Treg differentiation program. This notion would be consistent with our finding of a reduced T cell proliferative response in spleen. Moreover, more recently Song and coworkers reported that TGF-ß induced PD-L1 expression on DC, which in turn resulted in Foxp3^+^ Treg induction, but also in T cell apoptosis [51]. The tolerance-promoting effect of TGF-ß was demonstrated as important for the maintenance of the immunosuppressive and tolerance-promoting environment within the anterior chamber of the eye, termed anterior chamber-associated immune deviation (reviewed in [52]). In several autoimmune disease models antigen injected into this compartment was shown to be internalized by resident F4/80^+^ myeloid cells which subsequently induced Tregs in a complex interplay with other leukocytes. More recently, Farooq and coworkers demonstrated that injection of encephalitogenic antigens into the anterior chamber of the eye induced functionally active antigen-specific CD8^+^ Tregs [53]. Treg induction in vivo was also achieved by prior in vitro treatment of myeloid APCs with antigen plus TGF-ß2, followed by in vivo administration [54].

Our observation of unaltered frequencies of Foxp3^+^ Tregs in DNA vaccinated mice despite lower infiltration of Th1/Th17 cells into the CNS is in line with previous reports, which also documented the failure of i.m. DNA vaccination to increase Treg numbers, when the plasmid employed encoded MOGp91-108 with or without an immunoregulatory cytokine [55]. Other studies, on the other hand, reported on enhanced numbers of Tregs following i.m. administration of MOG-encoding DNA [39]. The different results are probably due to the usage of different immunization regimen, vector backbones and animal models.

Altogether, our findings suggest deletion of or anergy induction in MOG-specific CD4^+^ T cells in the periphery by DCs rendered tolerogenic following vaccination with MOG-encoding vectors combined with the TGF-ß1-encoding plasmid as the basis of inhibition of EAE pathology. This might result in reduced cell infiltration into the CNS resulting in lower EAE symptom scores.

The reason for the different effects of pCMV-IL10 and pCMV-TGFß in combination with pCMV-MOG on disease outcome is not clear. In the light of our hypothesis, the difference might possibly be attributed to the mechanism of action of the two immunomodulatory cytokines. While IL-10 is secreted, human DCs were reported to express TGF-ß in an inactive membrane-bound form (latency-associated peptide, LAP), from which active TGF-ß is released under the influence of diverse activation factors [56]. The authors demonstrated that surface-expressed TGF-ß reduced activation of naïve and effector T cells. In case of treatment of mice with pCMV-IL10 in combination with pCMV-MOG, the transfected keratinocytes secrete IL-10, but the amount of IL-10 does not appear to suffice to render those DCs in their neighborhood tolerogenic that have phagocytozed apoptotic vesicles harbouring MOG. In consequence, immunogenic MOG-presenting DCs would outnumber DCs that present MOG peptides at protolerogenic state due to direct transfection, thereby resulting in EAE pathology following EAE induction. In contrast, in case of treatment of mice with pCMV-TGFß combined with pCMV-MOG, apoptotic vesicles from transfected keratinocytes, that express MOG and membrane-bound TGF-ß1, are envisaged to render those DCs in their neighborhood tolerogenic, that have incorporated the vesicles. Hence, all or most MOG-presenting DCs would induce tolerance resulting in amelioration of EAE symptoms. This argument is strengthened by earlier findings that migratory DCs from skin transfer antigen to lymph node-resident DCs for presentation and T cell priming [57]. Along this line, surface-expressed TGF-ß might be transferred to resident DCs together with antigen. When, however, IL-10- or TGF-ß-encoding vectors are applied to the skin of mice in combination with pFscn-MOG, all directly transfected MOG-presenting DCs exert tolerogenic function.

A further explanation for the finding that the choice of promoter (CMV versus fascin1) used for MOG expression determined, whether coapplication of the IL-10 expressing vector yielded tolerance (fascin1 promotor) or not (CMV promotor), might be based on the level of MOG expression induced. The CMV promotor which is frequently used for transgene expression is known to strongly depend on NF-κB activity [58]. Fascin1 is upregulated by DC activation [59], but the promoter-regulating transcription factors have not been characterized yet. IL-10 via JAK signaling activates the transcription factor STAT3 which in turn modulates NF-κB activity via different mechanisms [60,61]. Therefore, it remains possible that IL-10 may differentially affect the fascin1 and the CMV promotor activities and thereby levels of antigen expression. More recently, persistent antigen presentation on a certain level was shown to be required for tolerance induction in EAE [62]. Hence, the level of promotor-dependent antigen expression may constitute a critical factor to promote tolerance which may be modulated by coexpressed transgenes.

Taken together, our findings suggest deletion of or anergy induction in MOG-specific CD4^+^ T cells in the periphery by DCs rendered tolerogenic following vaccination with MOG-encoding vectors combined with the TGF-ß1-encoding plasmid. As a consequence, the systemic Th17 and Th1 response in the context of EAE induction and the infiltration of activated Th17 and Th1 cells into the CNS are significantly reduced. This might result in lower EAE symptom scores. Our findings show that in the setting described, the fascin1 promoter proved comparable (coexpression of TGF-ß) and even superior (coexpression of IL-10) to the CMV promoter. It remains to be tested whether higher doses of DNA vaccine may imprint stronger tolerance. Moreover, additional work needs to address in more detail by which mechanisms preventive DNA vaccination with pFscn-MOG plus IL-10 or TGF-ß encoding vectors resulted in diminished Th1/Th17 responses, and thereby attenuated EAE progression. As stated above, induction of antigen-specific T cell anergy/apoptosis as well as Foxp3^--^ Tregs by tolerized DCs may contribute. In this regard, IL-10 and TGF-ß were reported before to suppress the production of GM-CSF as reviewed in [41]. GM-CSF, in turn, has been implicated in EAE pathogenesis, e.g. by regulating the inflammatory activity and differentiation of Th17 cells as well as promoting the infiltration of macrophages into the CNS. Reduced production of GM-CSF might therefore result in amelioration of EAE. Anyway, our study demonstrates that the fascin1 promoter due to its DC-focussed activity is an interesting alternative to ubiquitously expressed promoters for vaccination strategies.

## Supporting information

S1 Fig Gating strategy for analysis of adoptively transferred MACS-purified CFSE-labeled CD4^+^CD25^-^ 2D2/Thy1.1 T cells (CD90.1^+^) in mesenteric lymph nodes of recipients (CD90.2^+^) five days after DNA vaccination as described in Fig 1C. (TIF)

S2 Fig Detection of CNS-infiltrating immune cells and demyelination in cranial spinal cords. Histological sections of cranial spinal cords from mice vaccinated with either the control vector copGFP+CMV-GFP (upper row) or with a MOG-encoding plasmid in combination with CMV-TGFß (bottom row) were prepared on d16 after EAE induction as described in Fig 3. Sections were incubated with anti-CD3 (for T cells), anti-MAC3 (for macrophages/microglia) and anti-B220 (for B cells) antibodies. Incubation of sections with myelin-staining luxol fast blue (LFB) served to quantify EAE-induced demyelination. Scale bar = 40 μm.
(TIF)

S3 Fig Gating strategy for analysis of CNS-infiltrating cells (see Fig 4A). In the middle panel examples for differential course of EAE (upper graph: mild, lower graph: more severe) are shown. R2: CD11b^int^CD45.2^int^, R3: CD11b^neg/low^CD45.2^hi^, R4: CD11b^hi^CD45.2^hi^, R5: CD4^+^.
(TIF)

S4 Fig Gating strategy for analysis of CNS-infiltrating CD4^+^ T cells (see Fig 4B). R1: CD4^+^IL17^+^, R2: CD4^+^IFN-ϫ ^+^, R3: CD4^+^IL-17^+^IFN-ϫ^+^.
(TIF)

S5 Fig Gating strategy for assessment of CD4^+^Foxp3^+^ T cells (see Fig 6). (TIF)

## Abbreviations

CFA: complete Freund´s adjuvant
CFSE: carboxyfluorescein succinimidyl ester
CMV: cytomegalovirus
CNS: central nervous system
DC(s): dendritic cell(s)
EAE: experimental autoimmune encephalomyelitis
EGFP: enhanced green fluorescent protein
i.m.: intramuscular
IFN-ϫ: interferon gamma
Ig: immunoglobulin
IL: interleukin
MOG: myelin oligodendrocyte glycoprotein
MS: multiple sclerosis
PMED: particle-mediated epidermal delivery
TGF-ß: tumor growth factor ß
Th: T helper
Treg: regulatory T cell
