# Supplementary figures and images for: Inhibition of experimental autoimmune encephalomyelitis by tolerance-promoting DNA vaccination focused to dendritic cells
Source: PLoS One. 2018 Feb 6;13(2):e0191927. doi: 10.1371/journal.pone.0191927 (PMC5800700; doi:10.1371/journal.pone.0191927)

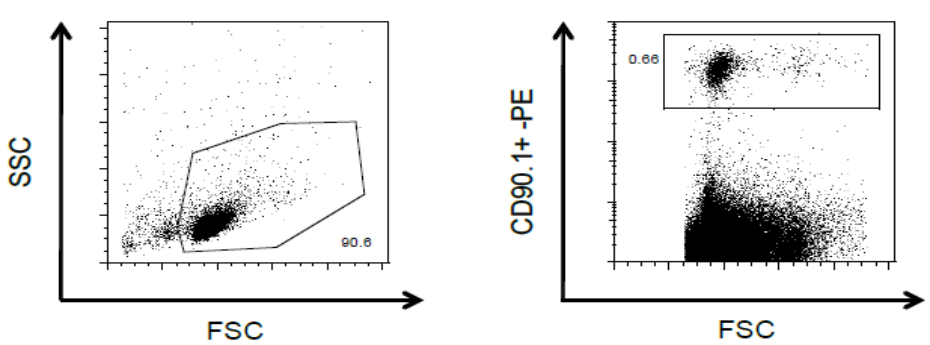

Supplement: S1 Fig — (TIF) [file pone.0191927.s001.tif]

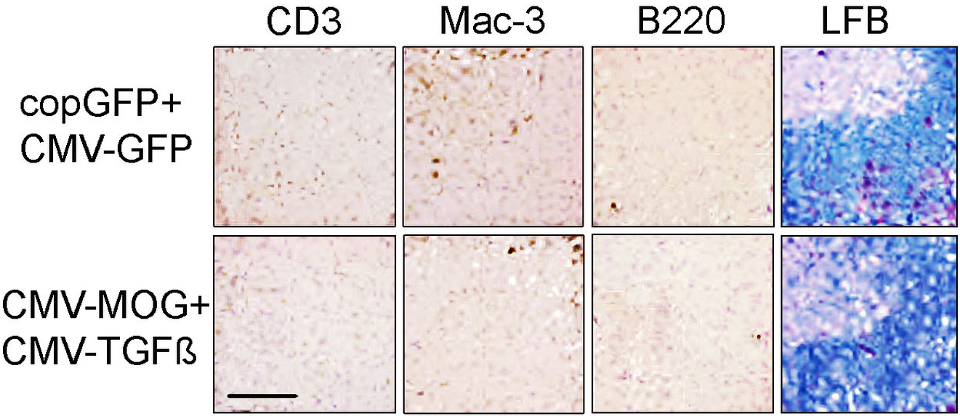

Supplement: S2 Fig — Histological sections of cranial spinal cords from mice vaccinated with either the control vector copGFP+CMV-GFP (upper row) or with a MOG-encoding plasmid in combination with CMV-TGFß (bottom row) were prepared on d16 after EAE induction as described in Fig 3. Sections were incubated with anti-CD3 (for T cells), anti-MAC3 (for macrophages/microglia) and anti-B220 (for B cells) antibodies. Incubation of sections with myelin-staining luxol fast blue (LFB) served to quantify EAE-induced demyelination. Scale bar = 40 μm. (TIF) [file pone.0191927.s002.tif]

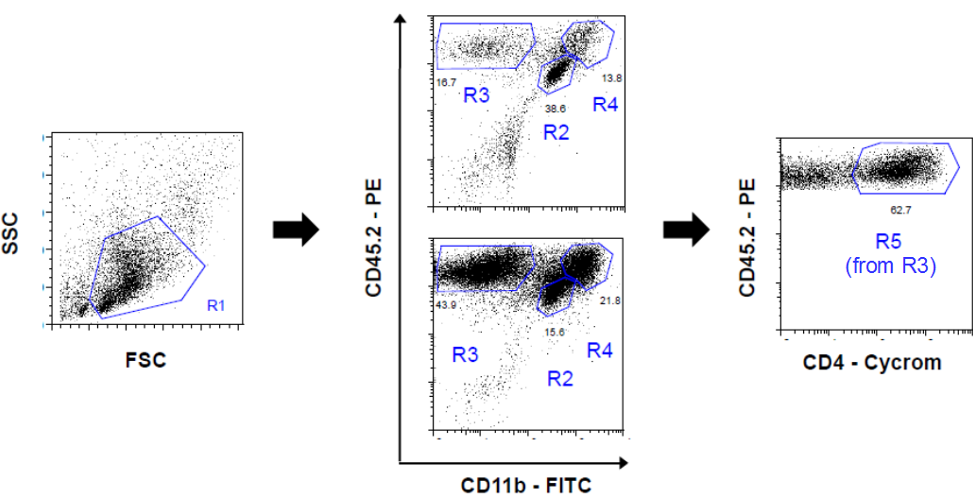

Supplement: S3 Fig — In the middle panel examples for differential course of EAE (upper graph: mild, lower graph: more severe) are shown. R2: CD11bintCD45.2int, R3: CD11bneg/lowCD45.2hi, R4: CD11bhiCD45.2hi, R5: CD4+. (TIF) [file pone.0191927.s003.tif]

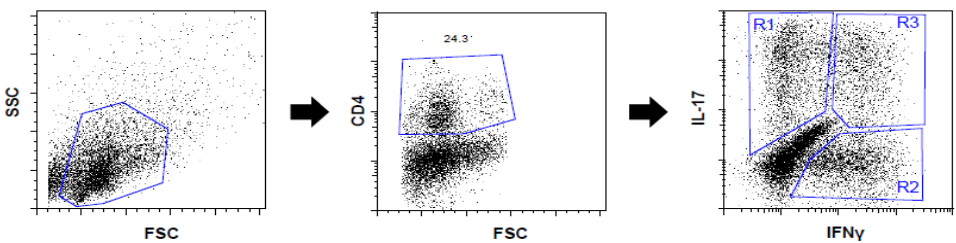

Supplement: S4 Fig — R1: CD4+IL17+, R2: CD4+IFN-ϫ +, R3: CD4+IL-17+IFN-ϫ+. (TIF) [file pone.0191927.s004.tif]

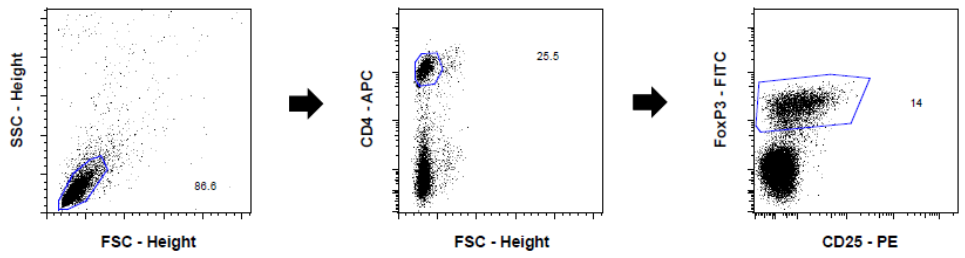

Supplement: S5 Fig — (TIF) [file pone.0191927.s005.tif]
